# The Influence of Traffic-Related Air Pollution (TRAP) in Primary Schools and Residential Proximity to Traffic Sources on Histone H3 Level in Selected Malaysian Children

**DOI:** 10.3390/ijerph18157995

**Published:** 2021-07-28

**Authors:** Nur Faseeha Suhaimi, Juliana Jalaludin, Suhaili Abu Bakar

**Affiliations:** 1 Department of Environmental and Occupational Health, Faculty of Medicine and Health Sciences, Universiti Putra Malaysia, Serdang 43400, Malaysia; nurfaseeha91@gmail.com; 2 Department of Occupational Health and Safety, Faculty of Public Health, Universitas Airlangga, Surabaya 60115, Indonesia; 3 Department of Biomedical Science, Faculty of Medicine and Health Sciences, Universiti Putra Malaysia, Serdang 43400, Malaysia; suhaili_ab@upm.edu.my

**Keywords:** traffic-related air pollution, primary schools, vulnerable population, indoor air quality, histone H3

## Abstract

This study aimed to investigate the association between traffic-related air pollution (TRAP) exposure and histone H3 modification among school children in high-traffic (HT) and low-traffic (LT) areas in Malaysia. Respondents’ background information and personal exposure to traffic sources were obtained from questionnaires distributed to randomly selected school children. Real-time monitoring instruments were used for 6-h measurements of PM_10_, PM_2.5_, PM_1_, NO_2_, SO_2_, O_3_, CO, and total volatile organic compounds (TVOC). Meanwhile, 24-h measurements of PM_2.5_-bound black carbon (BC) were performed using air sampling pumps. The salivary histone H3 level was captured using an enzyme-linked immunosorbent assay (ELISA). HT schools had significantly higher PM_10_, PM_2.5_, PM_1_, BC, NO_2_, SO_2_, O_3_, CO, and TVOC than LT schools, all at *p* < 0.001. Children in the HT area were more likely to get higher histone H3 levels (*z* = −5.13). There were positive weak correlations between histone H3 level and concentrations of NO_2_ (*r* = 0.37), CO (*r* = 0.36), PM_1_ (*r* = 0.35), PM_2.5_ (*r* = 0.34), SO_2_ (*r* = 0.34), PM_10_ (*r* = 0.33), O_3_ (*r* = 0.33), TVOC (*r* = 0.25), and BC (*r* = 0.19). Overall, this study proposes the possible role of histone H3 modification in interpreting the effects of TRAP exposure via non-genotoxic mechanisms.

## 1. Introduction

Large numbers of people reside and work in cities, including Malaysia, as offices, education centers, commercial sites, government hubs, and main transportation terminals are located here. Consequently, more people and buildings around the aforementioned area will cause increased traffic flow, especially during peak hours early in the morning and late in the evening, when most people travel to and from work, respectively. One of the detrimental environmental effects is the air pollution caused by roadside exposure in urban areas and children, who are susceptible to Traffic-Related Air Pollution (TRAP) exposure due to the location of schools and residential areas (within proximity of urban areas), will be affected.

Previous studies have proven that TRAP is associated with respiratory toxicity in different approaches, such as in vitro [1], in vivo [2], and epidemiology [3]. Generation of the reactive oxygen species (ROS) and oxidative metabolites, that trigger the oxidative stress in the cells located at the lung airways, are the main effects of the TRAP that leads to numerous respiratory health effects [4]. In addition, there is significant evidence on the epigenetic changes to the deoxyribonucleic acid (DNA) caused by TRAP [2,5]. Moreover, some studies have reported on the prevalence of respiratory symptoms in individuals who reside nearby high-traffic roads [6,7].

Epigenetic changes have been considered a link between TRAP and respiratory problems. The epigenetic study is evolving into becoming a promising novel path to determine the potential impacts of air pollution on the human DNA sequence, because some of these epigenetic mechanisms have been linked to diseases such as lung cancer. Studies have shown that the epigenetic modifications activated by environmental agents have unveiled histone modification levels; these changes, found among healthy respondents, are comparable to the observed epigenetic modifications found in patients whose conditions are induced by the same environmental agent [8,9]. Children are more susceptible to TRAP and, hence, their detoxification enzymes are less competent; subsequently, this may cause changes in chromatin structure or DNA, followed by epigenetic modifications [10].

It is crucial to understand the full extent of epigenetics, which is also an important component of the exposome. The exposome is a concept in environmental health that describes human exposure from pre-conception and onwards throughout a person’s lifespan [11]. Although epigenetic mechanisms are ideal molecular intermediates of environmental effects, the magnitude of individual environmental exposure that might be mediated through epigenetic mechanisms remains unclear and, hence, calls for more urgently needed research in this area [10]. Moreover, it is vital to understand the impacts that breathing these particles has on our epigenetic signature and our health, as air pollution continues to rise at an alarming rate. However, there are still limited research findings for histone modification, compared with other epigenetic mechanisms, such as DNA methylation, because their molecular pathways are not widely studied [12,13]. Therefore, this study was performed to investigate the association of TRAP with histone H3 modification.

## 2. Materials and Methods

### 2.1. Study Location

**Figure 1:**
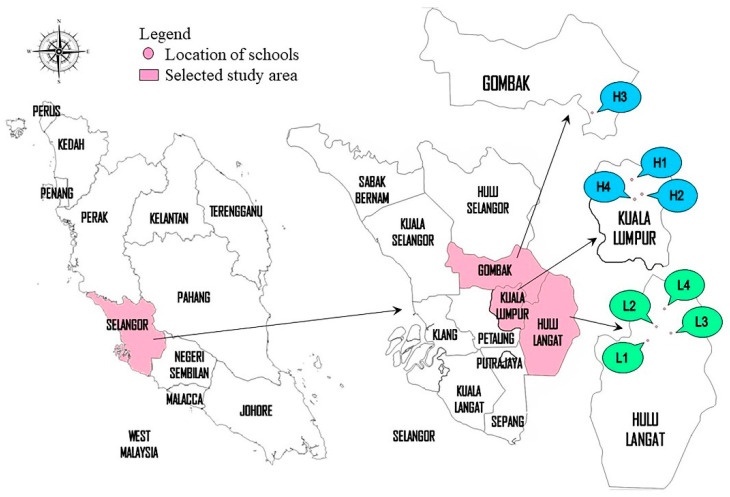
Locations of the selected primary schools.

The study was performed in Klang Valley, which is centred in Kuala Lumpur, and included its adjacent cities and towns in Selangor. Also known as the Greater Kuala Lumpur, Klang Valley is geographically defined by the Titiwangsa Mountains to the north and east, as well as the Strait of Malacca to the west. The TRAP zone is defined as 500 m on either side of highways with an average daily traffic (ADT) of ≥18,000 vehicles or 100 m on either side of major roads with an ADT of ≥15,000, ≥two lanes stretching several km, and a speed limit of >50 km/h [14]. The TRAP was measured at four primary schools located in the TRAP zone, which were categorized as the HT areas for this study. On the other hand, four primary schools in the low-traffic (LT) areas were determined from the location at a distance of more than 5 km away from nearby highways, major roadways, and industrial sites in Selangor (Figure 1). None of the schools in Kuala Lumpur was categorized in the LT sites, because these schools are located in the urban areas. A distance of 5 km was chosen to represent the average distance of traffic exposure [15,16]. These HT and LT sites are located in the same state to minimize confounding factors that might influence the outcome, such as sociodemographic and socioeconomic factors.

### 2.2. Study Population

The cross-sectional comparative study was performed between January and May 2019. The study sample included 248 primary school children, between 7 and 11, who fulfilled the inclusion and exclusion criteria. These respondents were calculated using a sample size calculation by Lemeshow et al. [17] after considering the 20% non-response rate and were selected randomly. Children with a history of doctor-diagnosed chronic respiratory diseases or allergies, unstable heart conditions, or severe respiratory infections were excluded from this research. Consent forms were distributed to be read and signed by parents or guardians. Only children who were given permission were included in the study.

### 2.3. Questionnaires

Standardized questionnaires from ISAAC and ATS (ATS-DLD-78-C) were adapted and developed to accommodate Malaysia’s environment. The questionnaires were filled in by parents or legal guardians of the children. Moreover, the questionnaires were translated from English to Malay and validated by a previous local study [18]. The pre-test of the questionnaire was conducted on at least 10% of the total respondents, which was among 29 respondents. The α value for the reliability test of Cronbach was deemed as acceptable for 0.8 > α ≥ 0.7.

### 2.4. Measurements of TRAP in Schools

The exposure to TRAP has been assessed either by direct measures of traffic or the measurement of air pollutant concentrations. The TRAP in this study, including particulate matter ≤10 µm in aerodynamic diameter (PM_10_), particulate matter ≤2.5 µm in aerodynamic diameter (PM_2.5_), particulate matter ≤1 µm in aerodynamic diameter (PM_1_), black carbon (BC), NO_2_, SO_2_, TVOC, and CO, were measured based on the method recommended in the manual of each instrument and previous studies. The measurements of PM_10_, PM_2.5_, and PM_1_ in classrooms were taken using a DustTrak DRX Aerosol Monitor (TSI, Shoreview, MN, USA; model 8534). The data were collected in real-time at each site for 6 h during the school session from 7.20 a.m. to 1.20 p.m. for a 1-min interval, which was done with the incorporation of a data logger. NO_2_, SO_2_, and CO were measured using an air quality monitor (Aeroqual, Auckland, New Zealand; model Series 500), a portable gas sensor. The TVOC was measured using a handheld VOC monitor (RAE Systems, San Jose, CA, USA; model PpbRAE 3000). These instruments also measured air pollutants in real-time and were accompanied by a data logger. The measurements of BC in PM_2.5_ were taken using a MiniVol Air Sampler (Airmetrics, Springfield, OR, USA; model 4.2), with samples on 47 mm quartz microfiber filters (Whatman, Marlborough, MA, USA; catalog number 1851-047). This battery-operated instrument was placed on its stand, on the ground, to sample ambient air at 5 L/min.

This cross-sectional study assessed air pollutants in multilevel classrooms from the ground to the first and second floors, because air pollutants mix to a different height in the school building. Hence, air pollutants measurements were varied from floor to floor, due to the deposition behaviors of air pollutants. For indoor monitoring, the instruments were positioned at the back of the classrooms (to minimize interference with everyday activities in the classroom); they were explicitly placed at 1.0 m above the floor at the back of the classrooms, away (at least 0.5 m) from the window, door, and obvious sources of potential contaminants (e.g., bookshelves), as adapted from a previous study [19]. Meanwhile, for outdoor monitoring, they were placed at the location closest to the traffic source (e.g., school fields and guard posts).

### 2.5. Gravimetric and Reflectometric Analysis of BC in Schools

The filter papers used were weighed pre- and post-exposure to determine the concentration in µg/m^3^. No pre-cleaning was involved for the filter papers. However, each filter paper was wrapped with aluminum foil and pre-baked at 500 °C for 4 h inside a furnace before sampling. Next, the filter media were kept in Petri pad dishes. The filter papers were stored for 48 h in a desiccator and below 25% RH before weighing to reduce water adsorption. The PM_2.5_ mass was measured by weighing the filter papers using a semi-micro analytical balance (A&D, San Jose, CA, USA; model GR-202) with 0.01 mg sensitivity. During the travel period, the filter papers were kept in an airtight container with silica gels. A total of 72 filters were collected, including 8 blanks (one for each school). The samples were kept in a desiccator before being analyzed. The calculation of 24-h PM_2.5_ measurements in schools is shown in Equations (1) and (2) below, following the manual by Airmetrics:

(1) $$V\;\left(m^{3}\right)=\frac{\frac{60\;\min}{h}\times \frac{1.7\;L}{\min}\times 24\;h}{\frac{1000\;L}{m^{3}}}$$

(2) $$PM\;\left(µg/m^{3}\right)=\frac{\left(W_{2}-W_{1}\right)}{V}\times 10^{6}$$

where *W*_1_ is the mass of filter paper before sampling (g), *W*_2_ is the mass of filter paper after sampling (g), and *V* is the volume of air sampled (m^3^).

BC samples were analyzed using a smoke stain reflectometer (Diffusion Systems, London, England; model EEL 43). This technique applies the measurement of the dark spots on the filter papers via their reflectance of white light, with a presumption of 100% reflection. Then, the absorbed light was transformed into real BC mass concentration after calculating several points throughout the filter paper, which were averaged. The blackness of a sample corresponded to BC content. The calculation of the BC measurement is shown below in Equation (3), as discussed previously, following ISO 9835 for BC standard [20]:

(3) $$BC\;\left(µg/m^{3}\right)=3.462\times 10^{9}\;{\left(\frac{A}{V}\;ln\left(\frac{R_{b}}{R_{s}}\right)\right)}^{2}+4.438\times 10^{5}\;\frac{A}{V}\;ln\left(\frac{R_{b}}{R_{s}}\right)$$

where *R_b_* is the intensity of light reflected from a clean filter, *R_s_* is the intensity of light reflected from a sampled filter, *A* is exposed filter area (m^2^), and *V* is the volume of air sampled (m^3^).

### 2.6. Saliva Collection and Processing

Respondents with permission were selected to undergo saliva collection to obtain an epigenetic biomarker of interest, histone H3. The method is effective and simple enough to facilitate among children. This procedure was carried out during school hours at rooms determined by the school management. The transmission of infectious diseases from saliva collection is likely, through active organisms suspended in droplets and aerosolized particles. Gloves were worn when handling the samples. After the saliva had been collected, the outer part of the containers was disinfected with alcohol wipes.

The saliva samples were collected in sterile 60 mL sample containers. Before saliva collection, the respondents had to wash their mouths with running water. Then, the respondents were asked to rub their tongue against the inside of the mouth for 15 s and expectorate saliva every 30 s into the container, on ice, until roughly 10 mL of saliva was collected. The containers were labeled with each respondent’s names, identification number, date, and time the sample was taken. Saliva samples were kept at 4 °C in an icebox with ice packs temporarily. Once the samples had reached the laboratory, the samples were aliquoted into sterile 1.5 mL microcentrifuge tubes (Eppendorf, Hamburg, Germany; catalog no. 0030120086). After that, the samples were centrifuged at 14,000× *g* for 15 min at 4 °C (MPW, Warszawa, Poland; model MPW-352R). The supernatant from each sample was frozen at −80 °C ultra-low temperature freezer (Sanyo, Osaka, Japan; model MDF 192) until extraction and quantification procedure.

### 2.7. Histone Extraction and Histone Modification Analysis

The approach to histone H3 modifications can be seen as a two-stage process: histone extraction and ELISA. The histones were extracted using a histone extraction kit (Epigentek, Farmingdale, NY, USA; catalog no. OP-0006-100), which used acid precipitation to isolate the highly basic histone from the samples. The protocol was adapted from the manufacturer’s protocol. Firstly, saliva samples were pre-lysed with a pre-lysis buffer on ice, centrifuged at 10,000 rpm for 1 min at 4 °C, and the supernatant was discarded. After that, the samples were lysed with a lysis buffer, incubated on ice for 30 min, and centrifuged at 12,000 rpm for 5 min at 4 °C. Finally, a pH balance buffer was added to the supernatant immediately. Circulating histone H3 was measured using a histone quantification kit (Epigentek, Farmingdale, NY, USA; catalog no. P-3091-96). The protocol was adapted from the manufacturer’s protocol.

Firstly, the samples, blank and positive controls, were run in duplicates in the 96-well ELISA plate. Then, the whole plate was covered and incubated at 37 °C for 60 min in an incubator. Histone H3 modified at specific sites was captured on the strip wells coated with an anti-histone H3 antibody. The reaction solution was removed, and the wells were washed 3 times with a wash buffer. Then, a detection antibody solution was added to each well. The whole plate was covered and incubated at room temperature for 60 min. The reaction solution was discarded, and the wells were washed 4 times with a wash buffer. Subsequently, a color development reagent was added to each well, and the plate was incubated at room temperature for 1 to 10 min (away from light). Finally, a stop solution was added to each well to cease the enzyme reaction. The absorbance or intensity of the color was measured on a microplate reader (Tecan, Männedorf, Switzerland; model Infinite M200) within 2 to 10 min at 450 nm, with an optional wavelength of 655 nm. A standard curve was generated with OD versus positive controls at each concentration point. The slope was determined as OD/ng. The amount of circulating histone H3 was computed using Equation (4), below:

(4) $$H3\;\left(\mathrm{ng}/\mathrm{mL}\right)=\frac{\left(Sample\;OD-Blank\;OD\right)}{Slope\times Sample\;Amount\;\left(µL\right)}\times 1000$$

### 2.8. Statistical Analyses

Data collected were analyzed using a Statistical Package for Science (SPSS) Version 23 (IBM, Armonk, NY, USA), RStudio Version 1.2.1335 (RStudio, Boston, MA, USA) and a GraphPad Prism 8 Version 8.4.2 (GraphPad Software, San Diego, CA, USA). The descriptive analyses were done to convey the important aspects of the data collected, including screening and organizing the data. Then, the collected data were analyzed at the univariate, bivariate, and multivariate levels accordingly.

### 2.9. Quality Control

The adapted questionnaires were pre-tested for 10% of the total respondents to ensure their validity and reliability. The α value for the reliability test (Cronbach) of 0.812 was deemed as acceptable for α ≥ 0.7. Each respondent was designated with a specific identification code to ensure anonymity and confidentiality.

Furthermore, the air monitoring equipment was calibrated before performing the measurements. Measurements of each air quality parameter were performed based on the method recommended in the manual of each instrument, as well as previous studies [21,22,23]. The equipment was kept clean, maintained, and checked regularly after use. They were also calibrated to zero before each measurement.

The PM_10_, PM_2.5_, and PM_1_ in schools were measured in the unit of µg/m^3^ using DustTrak DRX Aerosol Monitor for 6 h, based on the principle of light scattering. This instrument can detect aerosol concentration ranges from 0.001 to 150 mg/m^3^. Therefore, it can simultaneously measure both mass and size fraction concentration, corresponding to PM_10_, PM_2.5_, and PM_1_.

The BC in PM_2.5_ was measured gravimetrically in the unit of µg/m^3^ by using a MiniVol for 24 h, with samples on a quartz microfiber filter paper. The flow rate and elapse time of MiniVol were recorded before and after sampling. The two impactors of the MiniVol were cleaned and greased on the seventh sampling. These samples were then analyzed by using a smoke stain reflectometer. The blackness of a sample corresponds to the BC content after the calculation of several absorbed light points throughout the filter paper.

NO_2_, SO_2_, CO, and O_3_ were measured in the unit of parts per billion (ppb) using an Aeroqual S500, a portable gas sensor that accurately measures air pollutants in real-time. Sensors were mounted on an interchangeable cartridge that was installed to the monitor base. The sensors could be easily removed and replaced, so many gases can be measured using the same body. Each sensor head was equipped with an active sampling fan that guarantees a representative sample, increasing the measurement accuracy.

The presence of TVOC was assessed using a VOC monitor, which is a handheld gas monitor. It has a photoionization detector that provides instantaneous readings and ppb detection for gases, ranging from 1 ppb up to 10,000 ppb.

Meanwhile, the fresh saliva samples were kept at 2–7 °C in an icebox with ice packs for up to 24 h before being transported to a laboratory. Next, each respondent’s extracted saliva samples were preserved in a −80 °C freezer in the laboratory before carrying out the ELISA test. The histone H3 levels were calculated from the calibration curve.

## 3. Results and Discussion

### 3.1. Background Information

At the beginning of the study, 646 questionnaires were delivered, but only 536 questionnaires were returned, about 83%. After choosing only those who fulfilled the inclusion criteria, were granted permission by parents/guardians to participate in this research entirely, and did not withdraw from the biological sampling, there were a total of 248 children in this study, with 124 children from the high-traffic areas (and the other 124 children from the low-traffic areas). Table 1 shows the sociodemographic background of respondents from both areas. Overall, there was no significant difference between the two groups at *p* < 0.05 for all sociodemographic factors. According to primary school classification in Malaysia, 7–9 years old are classified as lower, while 10–12 years old are classified as upper primary school children. Nevertheless, 12-year-old children were excluded by the school management because they needed to prepare for the Primary School Evaluation Test.

Although the children were chosen according to their schools’ locations, whether in the HT or the LT region, it is arduous to restrict their residences’ locations. The types of properties and distances of residences from main roads, highways, and factories also guarantee that the difference between the two areas ruled each area’s exposure. A highway is a network of federal roads, while the main road is a network of state roads, as gazetted by the Malaysian Public Works Department [24]. It is shown that there were significant differences in the types of properties and the distances of residences from the highway between both groups at *p* < 0.001. Landed residences are residences on a whole piece of land, whereas strata residences are residences in a multi-story building, such as an apartment. In contrast, there was no significant difference in the distance of residences from main roads and factories between both groups at *p* < 0.05.

### 3.2. Estimation of Residential Proximity to Traffic Sources

Road proximity in this study was determined as the distance to the nearest major road (to estimate the TRAP), as previously applied [25,26]. Table 2 shows the number of respondents who reported the number of vehicles around their residences on weekdays and weekends. This information was obtained from the questionnaires. There were more vehicles reported around residential areas of the HT group than the LT group. Most of the LT group respondents (≥50%) reported that the number of vehicles around their residences was less than 100 per day, either during weekdays or weekends. However, there were different patterns for the HT group. Furthermore, cars and motorcycles were mainly reported to be <100 per day during weekends, while buses and lorries were mainly reported to be <100 per day, either during weekdays or weekends.

The exposure to TRAP varies in urban areas. The location and design of this study supported the assessment of the impact of TRAP in the Klang Valley region, where regional air pollution levels are relatively high [27,28]. This particular study area enabled a better focus on the variations in air quality related to localized TRAP. As proxies for TRAP exposure, residential proximity to the nearest major road and its traffic density were applied to investigate the relationships between TRAP exposure and health effects. Moreover, staying along heavily traveled roads is connected with an elevated risk of TRAP exposure [29].

Different approaches have been employed to determine the impact of residential TRAP on children. Self-reported traffic surveys are cost-effective to gather but inevitably subject to different perceptions and biases. Compared to objective quantitative exposures, self-reported traffic estimations, such as estimations of traffic counts around the residences or traffic annoyances, have demonstrated that subjective evaluations are robust in homogenous populations, particularly in urban areas [6]. While several previous research studies on respiratory diseases employing exposures to TRAP have been carried out, some studies have also shown associations using traffic indicators [30,31]. These indicators help to concentrate on air emissions linked explicitly to transportation.

Most of the HT group respondents live in strata houses, while most LT group respondents live in landed houses. The development within the urban vicinity has propelled more strata houses to accommodate the highly populated community. Low-cost flats are the most common residence type in the HT area, while traditional settlements are the most common type of residence in the LT area. To understand the source of outdoor pollutants exposure in residences, the location of each respondent’s residence was also assessed, based on information obtained from questionnaires. When economic growth and development were brought to urban areas, this affected the surrounding area and the recently built neighborhood within the vicinity, including establishing more road networks to transport human resources, supplies, and products. This finding agrees with a previous local study, which found a similar finding, whereby the location of their respondents’ residences from main roads was significantly different (*p* = 0.001) between the preschool children from the industrial and non-industrial group [32].

### 3.3. Concentrations of Air Pollutants in Schools

**Figure 2:**
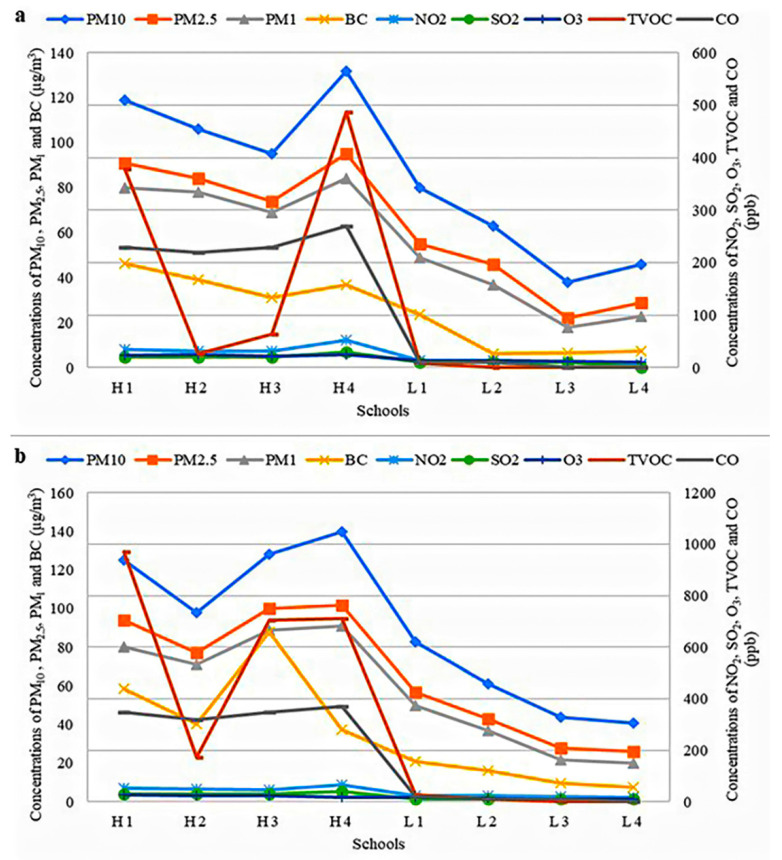
Average of (**a**) indoor and (**b**) outdoor air pollutants of the selected schools for five school days.

The indoor monitoring results are shown in Figure 2a. It was revealed that the exposure to TRAP was significantly higher for the HT group compared to the LT group. There were significant differences for all TRAP parameters between both groups at *p* < 0.05. On the other hand, the results for outdoor monitoring are displayed in Figure 2b. It was found that the exposure to TRAP was significantly higher for the HT group than the LT group. Only BC had 32 filter samples each for indoor and outdoor monitoring, due to its 24-h measurement duration, whereas the other parameters were measured for 6-h each session.

A local study discovered that BC was one of the major components of PM_2.5_, with a weight fraction of about 16% [33]. They conducted their studies at a site in Klang Valley, which was within a 15 km distance from all the schools in this study. Emissions with high BC typically represent incomplete combustion from motor vehicles, primarily diesel. When congestion occurs on roads, the speeds of vehicles and the following distance to other vehicles are reduced; hence, emissions from one vehicle can penetrate nearby vehicles [34]. BC has been associated with various health effects, such as cardiovascular disease, ischemic heart disease, asthma, and lung cancer [35]. A study in the Pearl River Delta region (China) had confirmed the possibility of carcinogenicity in children caused by BC exposure, by estimating an excess of 1.97 cancer cases per 10,000 children [36]. Meanwhile, a study in Chiang Mai (Thailand) had reported the health risks posed by BC was found comparable to a high number of passively smoked cigarettes, regarding the risk of percentage of lung function reduction of school-aged children [37]. There is no regulation for BC in Malaysia, but many PM_2.5_ reduction efforts should drive to decline BC exposure, particularly in the transportation scope.

Although a local study mentioned biomass burning is the second-highest PM contributor in Klang Valley [38], there was no haze occurrence during the sampling period. As for the outdoor sources caused by human activities, PM_10_ is commonly released from roadways, construction, and agricultural activities, whereas PM_2.5_ is commonly released from industrial processes and motor vehicles. A local study reported that PM_2.5_ in Klang Valley was contributed mainly by road dust and mixed secondary inorganic aerosol at about 32.4% to the PM_2.5_ mass [38]. Although PM_2.5_ continues to suspend in the air for several days and can be dispersed by winds across long distances from the initial source, PM_2.5_ may remain suspended in the air for quite some time [23]. Owing to its fine consistency, PM_2.5_ can be brought more deeply into the lungs and is deposited in the alveolar sacs [39]. The entry of PM_2.5_ into the respiratory system triggers a robust inflammatory response via phlegm, an indicator of mucus production [40]. The deposition of inhaled particles depends on the dimension of the airways; hence, there are differences in the dose deposited in various regions of the respiratory tract [41]. This mechanism explains why higher concentrations of particles may accumulate in children’s lower respiratory tract, compared to adults.

NO_2_ is classically regarded as a proxy for road traffic in outdoor city backgrounds, although it is not undoubtedly independent of other TRAP-related exposures [5]. A previous study had proven that NO_2_ is more dependent on traffic parameters than meteorological parameters [42] because NO that is emitted by vehicles undergoes a chemical reaction to generate NO_2_. Therefore, heavy traffic density around the schools in the HT area contributed to the high concentrations of NO_2_. NO_2_ has been implicated in the aetiology of oxidative damage [43]. By inducing the formation of the reactive oxygen species, NO_2_ causes cellular injury that triggers a cytokine response, which results in inflammation [44] and airflow obstruction [45].

Some schools (L1, L2, L3, and L4) reported very low SO_2_ concentration, which was below the limit of detection (LOD) at <10 ppb. This detection limit was defined by the lowest measurement detected by the instruments used in this study. In general, SO_2_ is generally emitted from industrial activities [46]. However, motor vehicles and, primarily, diesel-engined buses and lorries are likely to be the principal SO_2_ source at these selected schools. In a previous local study, sulfur from the combustion of engine fuel in petrol and diesel was found to add to the amount of SO_2_ in the environment, particularly in urban areas and suburban regions [47]. In contrast, fewer vehicles were traveling around the LT area, compared to the HT area. Moreover, there is no major industrial area, in both HT and LT areas, which removes the industrial source for SO_2_. SO_2_ has been associated with eye irritation, breathing problems, chest pains, heart disease, and respiratory mortality [48]. Inhalation of highly soluble SO_2_ will be metabolized into sulfites, which can interrupt DNA synthesis and activate chromosomal abnormality [3].

The concentration of ground O_3_ is highly dependent on the local emissions of O_3_ precursors (NO_x_ and VOC), solar intensity, and the local temperature [49]. Hence, the higher concentrations of VOC and NO_2_ in HT areas can influence O_3_ formation significantly, which were most likely sourced from vehicle emissions. This finding agrees with a previous local study, which reported that vehicular and industrial emissions might release higher O_3_ concentrations than the geographical proximity [50]. O_3_ can cause respiratory effects such as breathing difficulty and airway inflammation, which can later aggravate lung diseases and may even lead to premature deaths [51]. O_3_ contributes to cough by initiating action potentials in the nerve fibres, which are situated to sense the inhaled air environment [52].

Several schools (L1, L2, L3, and L4) recorded very low TVOC concentration (below LOD at <1 ppb). This detection limit was defined by the lowest measurement detected by the instruments used in this study. Although TVOC typically records higher concentration indoors, the findings in this study did not show similar outcomes because there was a limited source of TVOC, such as paints and floorings. The detected TVOC concentration was most likely contributed by traffic emissions outdoors, such as benzene, ethylbenzene, ethylbenzene, and xylene, as previous local studies had reported [49,53]. These four aromatics compounds are more commonly known as BTEX, resulting from traffic exhaust emissions and indoor sources. The classrooms at H1 and H4 schools were close to congested main roads and highways, especially during morning rush hours. Therefore, the outdoor TVOC released from vehicular emissions could have penetrated the classrooms. TVOC may irritate the eyes, nose, and throat, causing headaches and fatigue [54]. The TVOC are likely responsible for acute and chronic health effects, such as eye, nose, and throat irritation, as well as headache, fatigue, cardiovascular disease, blood-related diseases, and irreversible kidney failure [54,55].

Some schools (L1, L2, L3, and L4) reported very low CO concentration (below LOD at <10 ppb). This detection limit was defined by the lowest measurement detected by the instruments used in this study. The highest concentrations of CO were observed during peak hours, between 7.00 a.m. to 9.00 a.m., which was due to the massive traffic at those times. These findings are comparable with a local study, conducted in Kuala Lumpur, which discovered that higher CO emissions could be rooted in vehicles with higher cumulative travel mileage, engine modification, and no emission control device [56]. They also found that the lowest emission factors of CO were from vehicles with mileage below 20,000 km. CO is produced mainly by the incomplete combustion of vehicle fuel engines. Excessive TVOC and CO emissions are usually formed when the engine undergoes transmission conditions, such as during acceleration [57]. Driving around Klang Valley involves frequent accelerations and decelerations, due to the congestion of vehicles on the road. CO is recognized to be toxic at high concentrations and causes lung damage by competing with oxygen to bind to hemoglobin, thus reducing oxygen carrying efficiency [58]. Exposure to CO has been reported to lead to respiratory symptoms such as coughing, phlegm, and wheezing [59].

HT schools recorded higher average concentrations of air pollutants than LT schools because, in general, air pollutants were significantly higher at the HT schools than the LT schools, due to the high contribution of heavy traffic density. High-traffic density in Klang Valley is partly attributed to developments of housing complexes, underpasses, and flyovers [3]. School children in their indoor and outdoor environments can be the causes of air emissions in the classroom; for example, the movement of children inside and outside of the classroom contributes to the accumulation of PM in the classroom. Moreover, both anthropogenic activities and natural sources emit PM, whereas industrial activities or automobile exhaust release CO, SO_2_, and NO_2_ [46]. There are also percentages of sources other than traffic in TRAP, which complicates the segregation of the traffic impact.

### 3.4. The Trend of Air Pollutants in Nearby Continuous Air Quality Monitoring (CAQM) Stations

Batu Muda and Cheras stations were the nearest CAQM stations from the selected HT schools (and located in the city center of Kuala Lumpur). There was no CAQM station near the selected LT schools. Therefore, comparisons could only be made between primary data measured in HT schools and secondary data obtained from nearby CAQM stations. However, the comparison can only be made in terms of trend because there were differences in the type of instruments deployed between both measurements; hence, there were different flow rates.

Both stations show that PM_10_ and PM_2.5_ concentrations tend to peak around 9 a.m. For NO_2_, there is a very pronounced increase in concentrations during the peak morning rush hour. O_3_ shows very different behavior because O_3_ reacts with NO_2_. There is a very pronounced increase in O_3_ concentrations in the afternoon, between 2 and 3 p.m. CO and SO_2_ had similar daily trends. Just like NO_2_, CO showed a very pronounced increase in concentrations during the peak morning rush hour. These trends indicate very similar source origins, which are most likely traffic emissions.

Factors that impact air pollution distribution are wind speed (WS), wind direction (WD), ambient temperature (AT), relative humidity (RH), and solar radiation (SR). Meteorological conditions determine outflow strength and depend on megacity geographic location and the season for the period of emission [60]. Pollution Rose by RStudio is useful for considering pollutant concentrations by WD, or more specifically, the percentage time the concentration is in a particular range. This type of approach can be very informative regarding the air pollutant species. Figure S1 shows the dominance of north-easterly winds controlling the overall mean of PM_10_, PM_2.5_, NO_2_, SO_2_, O_3_, and CO concentrations at Batu Muda station. Meanwhile, most of the higher PM_10_, PM_2.5_, NO_2_, SO_2_, and CO concentrations at Cheras station are also associated with the easterly winds, as shown in Figure S2. Southern winds mainly influenced only O_3_ concentrations at Cheras station. These data presented the measurements at both CAQM stations throughout the year 2019.

Referring to Batu Muda station, WD is predominantly from the east and relatively low WS (0–2 m/s) throughout the year. WS is the highest in March (1.31 m/s) and the lowest in November (0.69 m/s). Both higher and lower concentrations of PM10 (Figure S1a), PM_2.5_ (Figure S1b), NO_2_ (Figure S1c), SO_2_ (Figure S1d), and CO (Figure S1f) at Batu Muda station are generally associated with northeast wind sectors. However, O_3_ concentrations at the Batu Muda station recorded non-similar findings; higher concentrations of O_3_ are generally associated with southeast wind sectors, while lower concentrations of O_3_ are generally associated with northeast wind sectors (Figure S1e).

Referring to Cheras station, WD is also predominantly from the east and relatively low WS (0–2 m/s) throughout the year. WS are the highest in August (1.11 m/s) and the lowest in February (0.88 m/s). Both higher and lower concentrations of PM_10_ (Figure S2a), PM_2.5_ (Figure S2b), NO_2_ (Figure S2c), SO_2_ (Figure S2d), and CO (Figure S2f) at Cheras station are generally associated with easterly wind sectors. However, O_3_ concentrations at Cheras station recorded non-similar findings; higher concentrations of O_3_ are generally associated with southerly wind sectors, while lower concentrations of O_3_ are generally associated with easterly wind sectors (Figure S2e).

All selected HT schools and both stations are in Klang Valley. The topography and climate of the Klang Valley have the potential to trap air pollutants [61]. Due to the valley topography and insufficient flushing along the valley that runs from the hilly terrain to the north, southeast, and the Malacca Straits in the west, pollutants are restricted [62]. It is experiencing worse air pollution problems due to increased traffic volume, urbanization, and industrial activities. Malaysia experiences four seasons, which are the northeast monsoon (NE) from December to March, inter-monsoon 1 (IM1) from April to May, southwest monsoon (SW) from June to September, and inter-monsoon 2 (IM2) from October to November [63]. On average, these monsoon seasons yield more rainfall than hot seasons with higher RH, lower SR, and lower AT. Klang Valley encountered the SW and IM1 during the study period (January–May).

H1, H2, H3, and H4 are located within 2.3 km (163° SE), 5.6 km (152° SE), 8.7 km (100° SE), and 5.9 km (168° SE) from the Batu Muda station. However, the wind that comes from these directions was <10% of the time during the study period except for H3, which had ~25%. Most higher concentrations of PM_10_, PM_2.5_, NO_2_, SO_2_, O_3_, and CO concentrations are associated with the NE. The dominance of north-easterly winds (46–75° WD) controlling the overall mean of air pollutants at Batu Muda station (Figure S1) is associated with the heavily populated outflow and massive regional traffic flow in North-eastern Kuala Lumpur, such as Wangsa Maju and Setiawangsa. The winds were calm most of the time throughout the study period, between 1.1 and 2.2%, despite monsoon seasons. It may get a little chilly after heavy rain, but Klang Valley is one of the areas least affected by monsoon winds coming either from the east or west [63].

H1, H2, H3, and H4 are located within 10.0 km (342° NW), 7.0 km (350° NW), 11.4 km (25° NE), and 6.5 km (337° NW) from the Cheras station. Nevertheless, the wind that comes from these directions was <5% of the time throughout the study period. The dominance of easterly winds (166–105° WD) was controlling the overall mean of air pollutants at Cheras station during NE monsoon, while the dominance of north-easterly winds (46–75° WD) controlling the overall mean of air pollutants at Cheras station during IM1 (Figure S2). These higher concentrations of air pollutants could be due to the transfer of air pollutants from the upwind area to the downwind area. The winds were calm most of the time throughout the study period, between 0 and 0.5% despite monsoon seasons.

### 3.5. Level of Histone H3 Modification

Circulating histones interact with membrane phospholipids by acting as mediators for organ injury, such as lungs [64]. Circulating histone H3 ELISA kit specific for humans was used to determine the concentration of histone H3 in saliva samples obtained from the respondents. This research is the first human study conducted in Malaysia to study exposure to air pollutants by associating them with histone H3 modifications among respondents, so there is no normal value for histone H3. Therefore, the median value was used as a cut-off point. Non-parametric analysis disclosed a significantly different level of histone H3 between the two groups. The median level was 885.10 (620.04) ng/mL in the HT group, which was significantly higher than that in the LT group at 623.41 (305.10) ng/mL (*z* = −5.13, *p* < 0.001).

**Figure 3:**
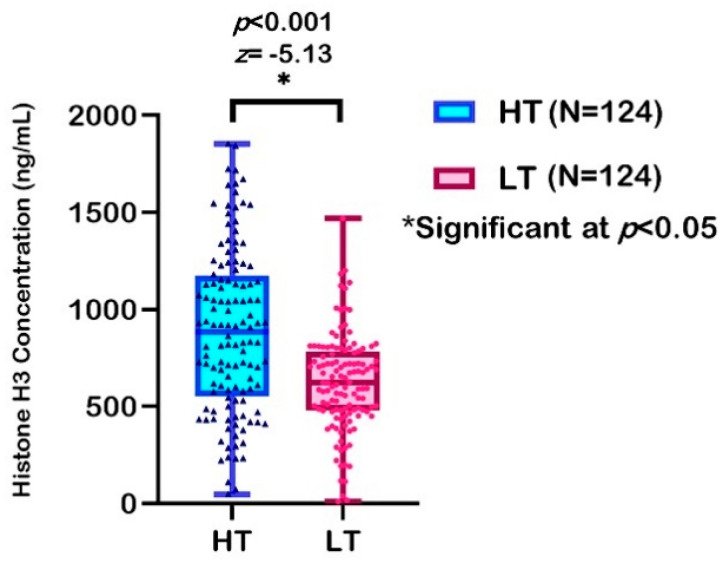
Expression profile of histone H3 modification between two groups of respondents.

A previous study reported that 76.5% of patients with severe blunt trauma (with circulating histone levels ≥50 µg/mL) experienced a respiratory breakdown, compared to 18.8% who had histone levels below this limit [64]. In another study, 75% of septic patients with histone levels ≥75 μg/mL had left ventricular dysfunction, compared with 8.3% of patients with histone levels <75 μg/mL [65]. Both studies used median data, which showed that a high level of circulating histones is possibly an early biomarker for lung damage and multiple organ dysfunction. In comparison to these two studies, the threshold histone H3 level obtained in the current study, among healthy children from HT and LT groups, was lower than the threshold level obtained in the previous studies. Figure 3 shows the expression profile of histone H3 level among both groups.

### 3.6. Relationships between Children’s Mode of Transport to Schools and Histone H3 Level

Traffic emissions lead to the accumulation of air pollutants in the atmosphere. Regardless of the school location, the comparisons between the children’s transport mode to schools and the histone H3 levels are reported in Table 3. These vehicles were further classified into “open vehicle” or “closed vehicle”; walk and motorcycle were categorized into “open vehicle”, whereas car and motorcycle were categorized into “closed vehicle”. Table 3 also displays the associations between the children’s mode of transport to schools and the histone H3 level. There was no significant association between the children’s mode of transport to school and the histone H3 level at *p* < 0.05. These findings could be due to the round-trip duration of time taken from home to school, which varies according to the type of vehicles, as shown in Table 3. Children who went to school by bus spent the most time on the road, followed by those who traveled by car, motorcycle, and walking. Children may be exposed to TRAP during the round-trip transport from their residences to schools, although the duration is short. Moreover, studies have proven high concentrations of air pollutants at pedestrian crossings, traffic intersections, and junctions [56,66].

Those who walked or rode motorcycles to schools were deemed to have higher exposure to TRAP during the round-trip travel period from home to school because they were exposed to inhaling pollutants from vehicle emissions. A previous study in Indonesia had testified that the time spent in and near vehicles had enormously contributed to the children’s daily exposure to TRAP [67]. Moreover, most Malaysian roads are choked with different vehicle types in the same lane [68]. Nonetheless, some of the users in closed vehicles tend to open the windows of the vehicles. Based on the findings in this study, 4 children (50.0%) who traveled by bus and 67 children (71.3%) who traveled by car to school reported open windows at least once during the round-trip travel period from home to school. These findings could be explained by higher exposures to air pollution experienced by those who used open transport vehicles than those who used closed transport vehicles. Besides, the health effects worsen when the same concentrations of air pollutants exposed to adults are also exposed to children.

### 3.7. Relationships between Respiratory Symptoms and Histone H3 Level

The prevalence of respiratory symptoms among school children has been reported earlier in another article [69]. Table 4 shows the associations between the reported respiratory symptoms and histone H3 level. There was no significant association between reported respiratory symptoms and histone H3 level at *p* < 0.05, except for cough. Cough was 3 times more likely to occur among those with a higher histone H3 level (*p* = 0.004, 95% CI = 1.39–6.58).

Exposure to air pollution generally causes oxidative stress [70], later regulating histone H3 and DNAm [71]. These epigenetic mechanisms modulate the gene expression by being responsive to changes in the environment of a cell, including the oxidative stress that leads to respiratory health effects [72]. Air pollutants, in the form of a foreign matter, could be the original elicitor that provokes cough; hence, it causes traumatic inflammation in the airways [73]. Asthma is one of the most prevalent inflammatory diseases, characterized by respiratory symptoms of varying severity. Many asthma-related genes undergo histone modifications [74]. Moreover, existing research suggests that epigenetic marks change gene expression in the lungs, which could be linked to respiratory diseases [75].

### 3.8. Correlations between TRAP Concentrations and Histone H3 Levels

Histones are particularly sensitive to oxidative stress, altering the molecular arrangement of these molecules and their natural working characteristics [76]. However, these histone modifications can either be caused directly or indirectly [77]. The high air pollution levels in the HT area could induce oxidative stress in the children and affect their histone H3 levels, as suggested by a previous study among newborns [78]. Air pollutants, particularly those that could freely access the alveoli, can increase reactive oxygen species production and affect the modification of histone H3 [9].

**Figure 4:**
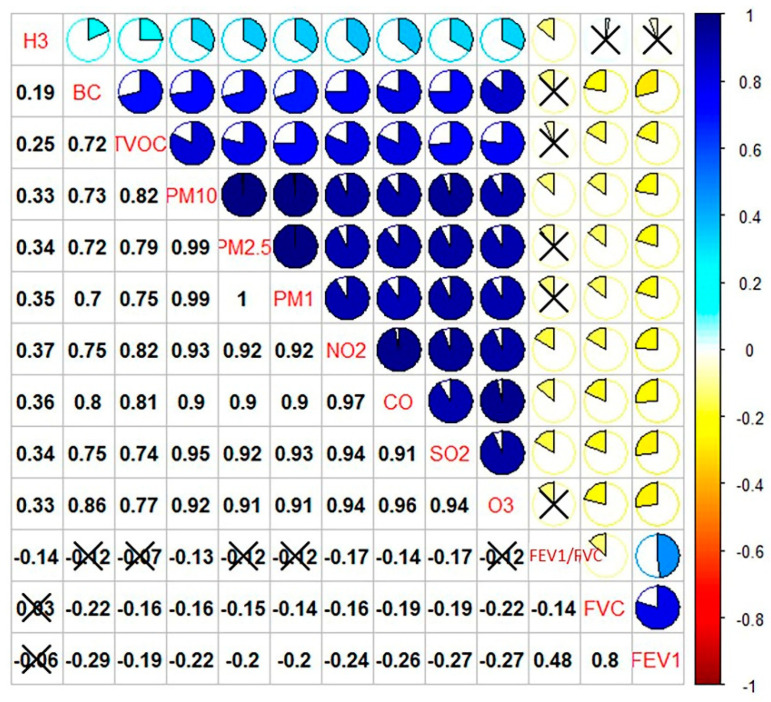
Correlations between TRAP concentrations and histone H3 level.

Figure 4 describes the correlation results between TRAP and the histone H3 level. The histone H3 level was positively and weakly correlated with concentrations of NO_2_ (*r* = 0.37), CO (*r* = 0.36), PM_1_ (*r* = 0.35), PM_2.5_ (*r* = 0.34), SO_2_ (*r* = 0.34), PM_10_ (*r* = 0.33), O_3_ (*r* = 0.33), TVOC (*r* = 0.25), and BC (*r* = 0.19). Minimal studies have examined the impacts of air pollutants on histone H3 modification; most of these studies were performed on occupational exposure [9,79]. Epigenetics has been suggested as one of the connections between air pollution exposure and respiratory health; for example, histone H3 modification in this study. Circulating histones function as the mediators of remote organ injury, inducing cellular calcium infusion by contact with membrane phospholipids [64]. No similar histone H3 level study in relation to air pollution exposure among children has been performed using ELISA. However, the results in this study agree with a cohort study, which investigated the adverse effects of air pollution exposure during gestation on circulating levels of histones among newborns [78]. There were positive associations between histone H3 and gestational exposure to PM_2.5_ and BC, with a 40.2% increase (95% CI, 24.1% to 58.3%, *p* < 0.001) and a 38.4% increase (95% CI, 6.2% to 80.3%, *p* = 0.003).

## 4. Conclusions

Although this study suggests that the concentrations of air pollutants in schools had weakly influenced the histone H3 level among the children, all nine air pollutants monitored were significantly higher among the schools and residences in the HT areas, compared to those in the LT area. The level of histone H3 was also significantly higher among the children in the HT group than those in the LT group. The children were exposed to TRAP from their transport mode to schools, the round-trip travel period from residences to schools, and their time spent at schools or homes. However, the extracted saliva samples had established uncertainty on which cell types were involved in the detection of the circulating histones; future research is needed to identify the cell types responsible for these findings.

Therefore, this study recommends maximizing the distance between future school sites and heavily traveled roadways to greatly reduce the level of exposure children may have to TRAP. Although it is better that schools are located further from heavily traveled roads, it is not easy to relocate existing schools, especially in Klang Valley, where the populations are increasing, but the available lands are reducing. Thus, road traffic density should be lessened around existing schools.

## Figures and Tables

**Table 1:** Background of respondents.

Variables	HT (*N* = 124)	LT (*N* = 124)	*χ* ^2^	*p*
	Number (%)			
Age				
7–9 years	59 (47.6)	63 (50.8)	0.26	0.611
10–11 years	65 (52.4)	61 (49.2)		
Gender				
Boy	65 (52.4)	53 (42.7)	2.33	0.127
Girl	59 (47.6)	71 (57.3)		
Types of Residence				
Landed	53 (42.7)	112 (90.3)	63.04	<0.001 *
Strata	71 (57.3)	12 (9.7)		
Distance of Residence from Highways				
<500 m	77 (62.1)	7 (5.6)	88.21	<0.001 *
≥500 m	47 (37.9)	117 (94.4)		
Distance of Residence from Main Roads				
<500 m	106 (85.5)	107 (86.3)	0.033	0.855
≥500 m	18 (14.5)	17 (13.7)		
Distance of Residence from Factories §				
<5 km	12 (9.7)	4 (3.2)	4.28	0.070
≥5 km	112 (90.3)	120 (96.8)		

* Significant at *p* < 0.05; § By *χ*^2^ test with Yates’ correction for expected value < 5.

**Table 2:** Number of vehicles reported by respondents around their residences on weekdays and weekends.

Variables	HT (*N* = 124)	LT (*N* = 124)	*χ* ^2^	*p*
	Number (%)			
Weekdays				
Car				
<100 vehicles/day	53 (42.7)	91 (73.4)	23.91	<0.001 *
≥100 vehicles/day	71 (57.3)	33 (26.6)		
Bus §				
<100 vehicles/day	109 (87.9)	122 (98.4)	9.09	0.003
≥100 vehicles/day	15 (12.1)	2 (1.6)		
Lorry				
<100 vehicles/day	111 (89.5)	118 (95.2)	2.79	0.095
≥100 vehicles/day	13 (10.5)	6 (4.8)		
Motorcycle				
<100 vehicles/day	59 (47.6)	85 (68.5)	11.19	0.001 *
≥100 vehicles/day	65 (52.4)	39 (31.5)		
Weekends				
Car				
<100 vehicles/day	69 (55.6)	89 (71.8)	6.98	0.008 *
≥100 vehicles/day	55 (44.4)	35 (28.2)		
Bus §				
<100 vehicles/day	109 (87.9)	122 (98.4)	9.09	0.003
≥100 vehicles/day	15 (12.1)	2 (1.6)		
Lorry §				
<100 vehicles/day	112 (90.3)	120 (96.8)	3.27	0.070
≥100 vehicles/day	12 (9.7)	4 (3.2)		
Motorcycle				
<100 vehicles/day	71 (57.3)	88 (71.0)	5.07	0.024 *
≥100 vehicles/day	53 (42.7)	36 (29.0)		

* Significant at *p* < 0.05; § By *χ*^2^ test with Yates’ correction for expected value < 5.

**Table 3:** Relationships between children’s mode of transport to schools and histone H3 level.

Variables	Duration (minute)				High Histone H3 Level, *N* (%)	OR	95% CI
	Min	Max	Mean	SD			
Comparisons							
Car (*N* = 94)	2	60	14.7	12.7	48 (51.1)		
Bus (*N* = 8)	5	30	15.9	8.3	6 (75.0)		
Walk (*N* = 22)	2	10	6.9	3.4	15 (68.2)		
Motorcycle (*N* = 124)	1	30	10.0	6.1	50 (40.3)		
Associations							
Open (*N* = 146)					54 (52.9)	1.40	0.84–2.33
Closed (*N* = 102)					65 (44.5)		

**Table 4:** Associations between reported respiratory symptoms and histone H3 level.

Respiratory Symptoms	Histone H3 OR (95% CI)
Cough	3.0 (1.39–6.58) *
Phlegm	2.8 (0.96–8.07)
Wheezing	2.1 (0.69–6.30)
Chest Tightness	1.3 (0.30–6.14) §

*N* = 248; * Significant at *p* < 0.05; § By *χ*^2^ test with Yates’ correction for expected value < 5.

## Data Availability

Not applicable.

## Supplementary Materials

The following are available online at https://www.mdpi.com/article/10.3390/ijerph18157995/s1, Figure S1: Pollution rose of (a) PM_10_, (b) PM_2.5_, (c) NO_2_, (d) SO_2_, (e) O_3_, and (f) CO in Batu Muda station in 2019; Figure S2: Pollution rose of (a) PM_10_, (b) PM_2.5_, (c) NO_2_, (d) SO_2_, (e) O_3_, and (f) CO in Cheras station in 2019.
