# Supplementary figures and images for: The Influence of Traffic-Related Air Pollution (TRAP) in Primary Schools and Residential Proximity to Traffic Sources on Histone H3 Level in Selected Malaysian Children
Source: Int J Environ Res Public Health. 2021 Jul 28;18(15):7995. doi: 10.3390/ijerph18157995 (PMC8345469; doi:10.3390/ijerph18157995)

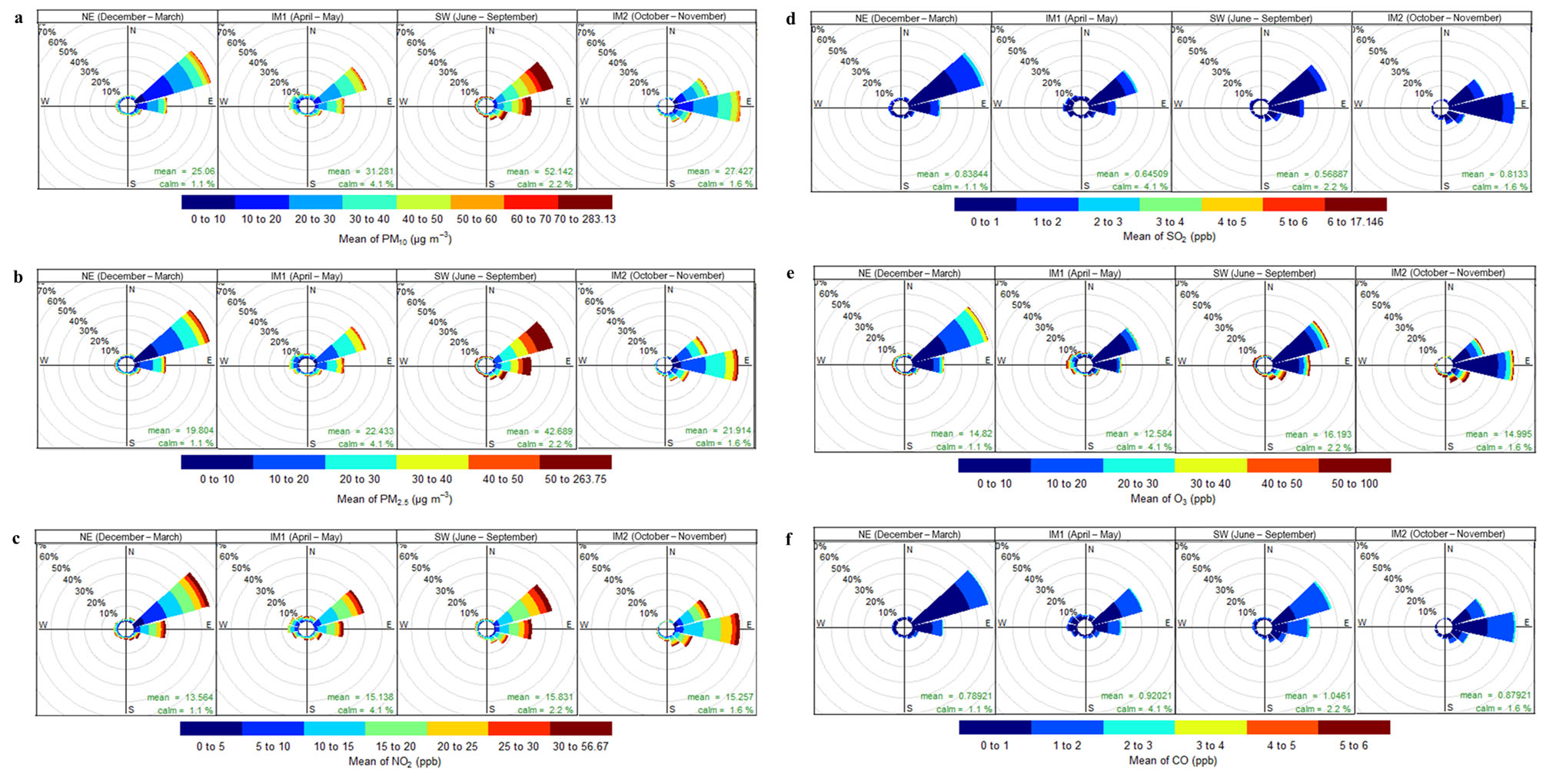

Supplement: Supplementary file 1 [file ijerph-18-07995-s001.zip › FSJJSA_FigureS1.png]

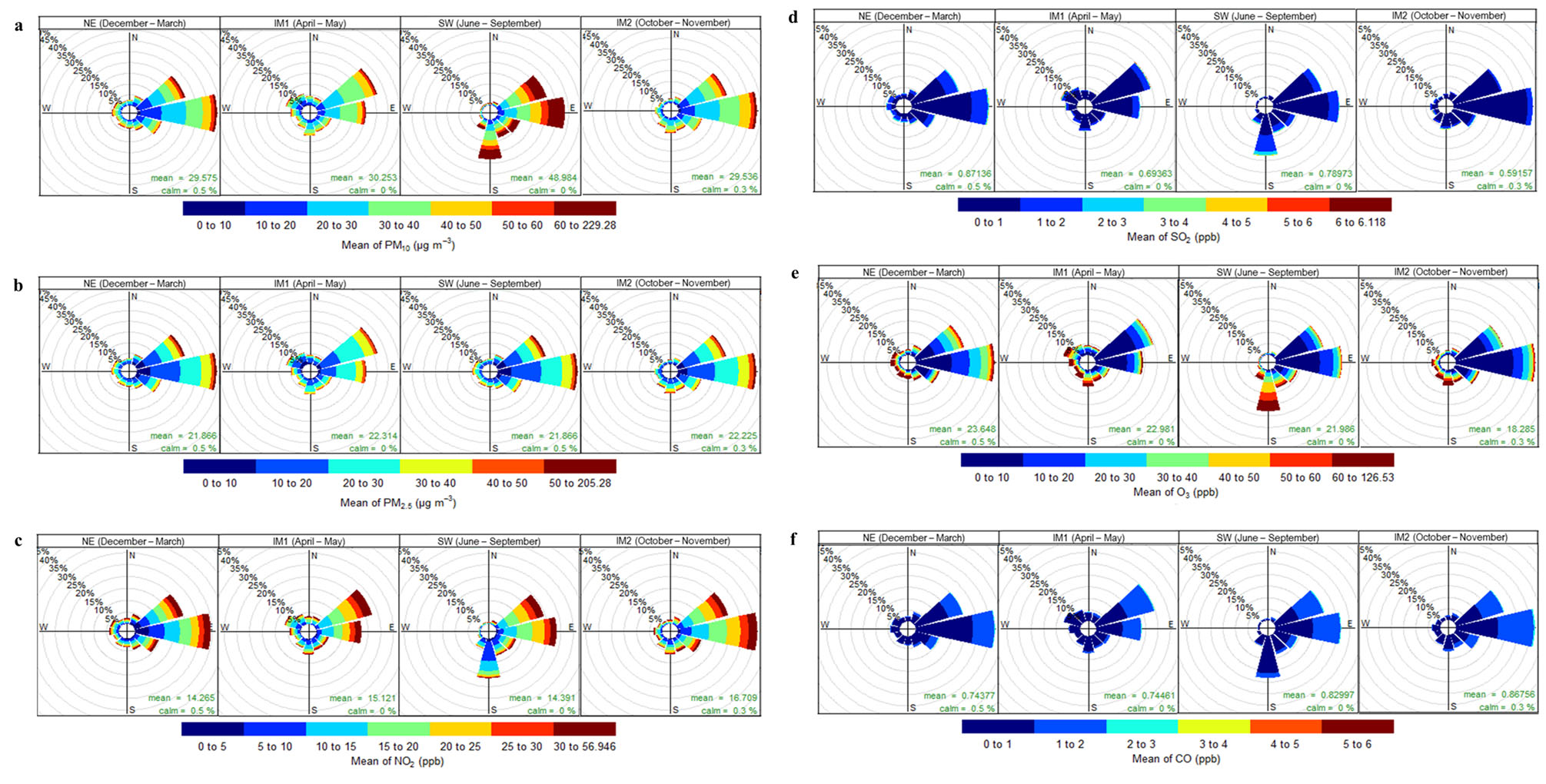

Supplement: Supplementary file 1 [file ijerph-18-07995-s001.zip › FSJJSA_FigureS2.png]
